# Protective Immune Responses Generated in a Murine Model Following Immunization with Recombinant *Schistosoma japonicum* Insulin Receptor

**DOI:** 10.3390/ijms19103088

**Published:** 2018-10-09

**Authors:** Hong You, Marina Harvie, Xiaofeng Du, Vanessa Rivera, Ping Zhang, Donald P. McManus

**Affiliations:** 1 Molecular Parasitology Laboratory, QIMR Berghofer Medical Research Institute, Brisbane QLD4006, Queensland, Australia; mcgharvie@gmail.com (M.H.); Xiaofeng.Du@qimrberghofer.edu.au (X.D.); Vanessa.Rivera@qimrberghofer.edu.au (V.R.); 2 Translational Cancer Immunotherapy Laboratory, QIMR Berghofer Medical Research Institute, Brisbane QLD4006, Queensland, Australia; Ping.Zhang@qimrberghofer.edu.au

**Keywords:** *Schistosoma japonicum*, insulin receptor, triose-phosphate isomerase, vaccine, murine model

## Abstract

There is a pressing need to develop vaccines for schistosomiasis given the current heavy dependency on praziquantel as the only available drug for treatment. We previously showed the ligand domain of the *Schistosoma japonicum* insulin receptor 1 and 2 (rSjLD1 and 2) fusion proteins conferred solid protection in mice against challenge infection with *S. japonicum*. To improve vaccine efficacy, we compared the immunogenicity and protective efficacy of rSjLD1 on its own and in combination with *S. japonicum* triose-phosphate isomerase (SjTPI), formulated with either of two adjuvants (QuilA and montanide ISA 720VG) in murine vaccine trials against *S. japonicum* challenge. The level of protection was higher in mice vaccinated only with rSjLD1 formulated with either adjuvant; rSjTPI or the rSjTPI-rSjLD1 combination resulted in a lower level of protection. Mirroring our previous results, there were significant reductions in the number of female worms (30–44%), faecal eggs (61–68%), liver eggs (44–56%), intestinal eggs (46–48%) and mature intestinal eggs (58–63%) in the rSjLD1-vaccinated mice compared with the adjuvant only groups. At 6-weeks post-cercarial challenge, a significantly increased production of interferon gamma (IFNγ) in rSjLD1-stimulated splenic CD4^+^ T cells was observed in the rSjLD1-vaccinated mice suggesting a Th1-type response is associated with the generated level of protective efficacy.

## 1. Introduction

Schistosomiasis remains one of the most prevalent, insidious and serious of the tropical parasitic diseases with some200 million people in 76 countries infected [1]. The antischistosomal praziquantel (PZQ) is cheap, effective and widely available, but drug treatment does not prevent reinfection. Thus, in spite of the wide-scale use of PZQ in the past 40 years, the numbers of infected individuals, especially in sub-Saharan Africa, remain at an unsatisfactorily high level [2]. In addition, drug-resistant parasites may evolve, mass drug administration programs are unsustainable, and highly protective anti-schistosome vaccines are not yet available. Recently, modelling studies have shown that bovines are major animal reservoir hosts for *Schistosoma japonicum* in the Peoples’ Republic of China [3] and the Philippines [4], contributing 90% schistosome egg contamination in the environment. Indeed, recent studies in the Philippines have indicated high schistosome prevalence, between 70–100%, in the buffalo population in endemic areas [4]. Mathematical modeling has predicted that a schistosomiasis japonica vaccine, able to reduce faecal egg output by 45% in water buffalo (responsible for 75% of disease transmission), in conjunction with PZQ treatment and other control measures, will lead to a substantial reduction in transmission leading to elimination [5]. This framework of transmission and elimination underpins efforts to develop a veterinary transmission blocking vaccine targeting fecundity and/or egg viability as it provides a realistic approach for the control of *S. japonicum* [3].

It is striking that adult schistosomes take up considerable quantities of glucose, consuming their dry weight of this sugar from their mammalian hosts every 5 h [6]. This is to provide energy for growth, development, pairing, maturation and egg production. Interrupting or blocking glucose uptake and metabolism, leading to decreased ATP synthesis, would result in the starvation of worms via a reduced supply of energy, thereby limiting and retarding these vital processes. Two types of insulin receptors have been isolated from *S. japonicum* (SjIR1 and SjIR2) [7]. These SjIRs induce a high level of immunological cross reactivity, they share similar functional properties and can bind parasite insulin-like peptide or human insulin, modulating the process of glucose uptake from host blood into the worm [8]. SjIR1 is present on the internal epithelium and tegument basal membrane of adult worms; SjIR2 is present in the parenchyma of males and the vitelline tissue of females, which occupies 82% of female tissue. Immunisation of mice with the L1 subdomain (insulin binding domain) of the SjIR2 (SjLD2) fusion proteins induced a significant retardation in worm growth (~42% reduction in worm length) and depressed fecundity (56–67% faecal egg reduction) [9] against a challenge with 34 cercariae, emphasising their potential as transmission blocking vaccine candidates.

Several studies have demonstrated that multivalent vaccines can induce higher protection against schistosome infection than antigens tested individually [10,11]. Aiming to improve the vaccine efficacy of SjIR, we used a multivalent vaccine approach, combining SjIR with another encouraging anti-schistosome vaccine candidate, triose phosphate isomerase (SjTPI), conjugated with two different adjuvants (QuilA and montanide ISA 720VG), and determined protective efficacy against a challenge *S. japonicum* infection. We hypothesised this multivalent vaccine targets simultaneously two key pathways of the worm’s energy supply: (i) by blocking the binding of the SjIR with host/parasite insulin, thereby reducing glucose uptake from the mammalian host; and (ii) by inhibiting the activity of SjTPI, the key enzyme in glycolysis and ATP generation following glucose uptake by the parasite from host blood. A DNA vaccine encoding SjTPI has been tested to prevent *S. japonicum* infection; the vaccine reduced worm burdens in mice (28%) [12], pigs (48%) [13] and water buffaloes (48–52%) [5,14] by inducing specific Th1-biased immune responses. SjTPI is located in most cells of the adult worm and on the membrane surface of the newly transformed schistosomulum, the parasite stage in mammalian hosts the most likely target of anti-schistosome vaccines [3].

The choice of an appropriate adjuvant aiding the stimulation of the relevant immune response and to increase the protective efficacy of a candidate vaccine antigen is important. Based on the high immunological cross reactivity between SjLD1 (L1 subdomain of the SjIR1) and SjLD2 and the fact that SjLD1 is located on the *S. japonicum* tegument surface—a likely prime target for immunological control—we tested the vaccine efficacy of rSjLD1 and SjTPI, both on their own and in combination, formulated separately with 2 different adjuvants (QuilA and ISA 720VG), in murine vaccine/challenge experiments with *S. japonicum.* In each case, the immunological profiles generated were assessed.

## 2. Results 

### 2.1. Protective Potential of SjLD1

The highest protective efficacy against *S. japonicum* challenge was observed in mice vaccinated with rSjLD1 formulated with QuilA or ISA 720, compared with mice immunised with rSjTPI or rSjLD1 + rSjTPI. Formulated with QuilA, rSjLD1 significantly induced a reduction in both female and male worm number, a reduction in faecal eggs, decreased liver and intestinal egg numbers, and a reduction in the number of mature intestinal eggs, compared with the adjuvant control group (Table 1 and Figure S1). Formulated with ISA720, rSjLD1 induced a reduction in female worm numbers, a reduction in faecal eggs, reductions in the numbers of liver and intestinal eggs, and a reduction in the number of mature intestinal eggs, compared with the ISA720 adjuvant control group (Table 1 and Figure S2). rSjLD1 conjugated with ISA720 also depressed worm growth resulting in a decrease in the lengths of female and male worms. In contrast, rSjTPI formulated with QuilA significantly induced a reduction in the number of mature intestinal eggs only (Table 1 and Figure S1) compared with the adjuvant only group. Formulated with ISA720, rSjTPI elicited reductions in female and male lengths, and a reduction in the number of mature intestinal eggs compared with the ISA720 adjuvant control group (Table 1 and Figure S2). The rSjLD1 + rSjTPI combination resulted in reductions in liver and intestinal eggs the stunting of adult worms and a decrease in mature intestinal eggs in vaccinated mice compared with adjuvant controls (Table 1 and Figure S2).

#### 2.1.1. Specific Antibody Titres

Specific antibody (IgG, IgG1, IgG2a, IgG2b, IgG2c and IgG3) titres in the sera of the different groups of vaccinated mice prior to cercarial challenge (at 2 weeks after the third injection) and prior to perfusion (at 6 weeks post-challenge with cercariae) are shown in Table S1. Prior to challenge, mice vaccinated with rSjLD1 or rSjTPI generated high levels of specific anti-rSjLD1 or anti-rSjTPI IgG, IgG1, IgG2a, IgG2b and IgG2c antibodies with comparatively lower IgG3 titre. The titres of all antibodies induced by immunisation with rSjLD1-QuilA were higher than those elicited in mice vaccinated with rSjLD1-ISA, with the exception of IgG3 antibodies which were similar in level in both trials. In contrast, mice vaccinated with rSjTPI-ISA induced higher levels of specific anti-rSjTPI IgG and IgG1, lower levels of IgG2b and IgG2c compared with those vaccinated with rSjTPI-QuilA; IgG2a antibody titres were similar in the two trials. Mice vaccinated with the combined rSjLD1 + rSjTPI conjugated with QuilA showed comparatively higher titres of specific anti-rSjLD1 IgG, IgG1, IgG2a, IgG2b, IgG2c and IgG3 antibodies, but lower titres of specific anti-rSjTPI IgG, IgG1, Ig2b antibodies, compared with mice immunised with the rSjLD1 + rSjTPI combination conjugated with ISA.

**Figure 1:**
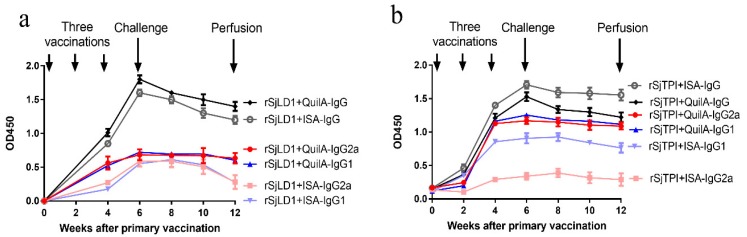
Kinetics of specific IgG and IgG1 and IgG2a antibody isotypes induced in mice immunised with the rSjLD1 and rSjTPI vaccine, respectively, conjugated with QuilA or ISA720. (**a**) Anti-rSjLD1 specific IgG, IgG1 and IgG2a antibody isotype levels (OD450) in mice vaccinated with rSjLD1 + QuilA or rSjLD1 + ISA and (**b**) Anti-rSjTPI specific IgG, IgG1 and IgG2a antibody isotype levels in mice vaccinated with rSjTPI + QuilA or rSjTPI + ISA are shown over the 12 weeks after the primary vaccination and include time points at week 0, weeks 2 and 4 after the primary vaccination (2 boosts), week 6 (just prior to challenge with *S. japonicum* cercariae) and week 12 (just prior to perfusion).

At 6 weeks post-challenge, the specific antibody titres measured in the sera of the vaccinated mice had declined but were still maintained at a high level (Table S1) with a similar pattern of antibody response shown prior to challenge. The kinetics of specific IgG, and IgG1 and IgG2a antibody isotypes against rSjLD1 and rSjTPI induced in mice immunised with the rSjLD1 and rSjTPI vaccine, respectively, conjugated with QuilA or ISA720 over a 12 weeks period are shown in Figure 1 and Table S2. Anti-rSjLD1 IgG antibodies in animals vaccinated with rSjLD1-QuilA or rSjLD1-ISA720 peaked at week 6 and then dropped over the next 6 weeks until the time of perfusion. Anti-rSjLD1 IgG1 and IgG2a antibodies rose and then plateaued at week 6. In contrast, both IgG1 and IgG2a levels elicited in mice vaccinated with rSjLD1-ISA dropped after challenge and thereafter until week 12 at the time of perfusion (Figure 1a). Anti-rSjTPI IgG antibodies in mice vaccinated with rSjTPI-QuilA or rSjTPI-ISA720 peaked at week 6 (at week 8 for IgG2a antibody in mice vaccinated with rSjTPI + ISA) and then dropped over the following weeks until the time of perfusion. Both IgG1 and IgG2a levels elicited in mice vaccinated with rSjTPI-QuilA rose and then plateaued at week 4 and remained at a similar level until the time of perfusion. In contrast, both IgG1 and IgG2a levels elicited in mice vaccinated with rSjTPI-ISA dropped by 2 weeks after challenge (Figure 1b). However, rSjTPI conjugated with ISA elicited a higher total IgG response compared with that conjugated with QuilA, whereas QuilA conjugated with rSjLD1 showed a better IgG response than ISA adjuvant.

#### 2.1.2. Cytokine Production

Splenocytes isolated from vaccinated and control mice were stimulated with SEA, SWAP, rSjLD1 and rSjTPI in vitro after which intracellular cytokine staining and multi-colour flow cytometry were undertaken. The splenic T cell response to these antigens was determined as a proportion of IL-4^+^ and IFNγ^+^ cytokine-producing cells.

##### Cytokine Responses in Mice Vaccinated with rSjLD1, rSjTPI and the rSjLD1 + rSjTPI Combination Adjuvanted with ISA720

**Figure 2:**
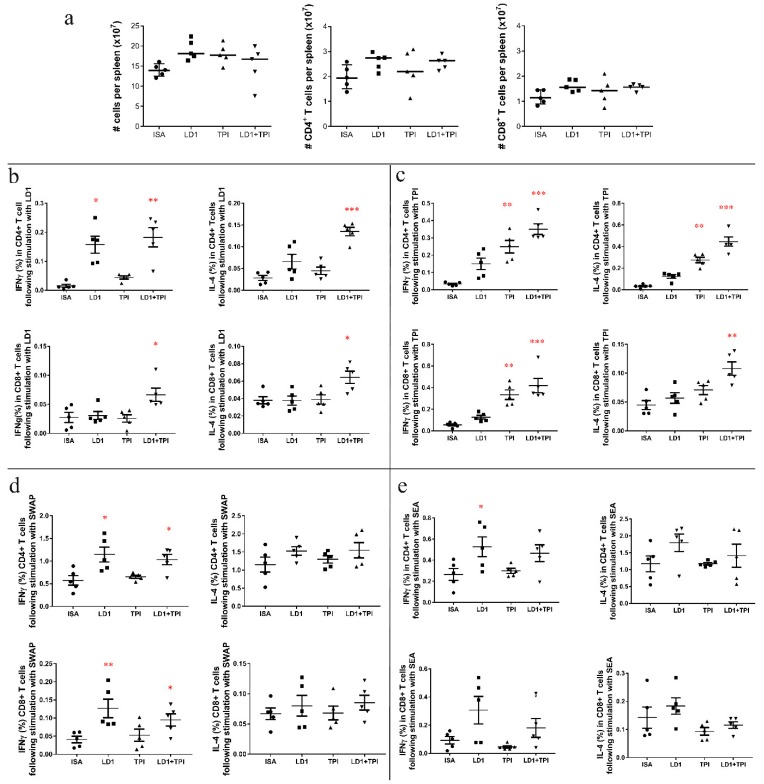
The cytokine profiles of splenocytes recovered from mice vaccinated with rSjLD1, rSjTPI and rSjLD1 + SjTPI adjuvanted with ISA720 at 6 weeks post-challenge. Splenocytes isolated from vaccinated and adjuvant control mice were stimulated with medium (as a control), rSjLD1, rSjTPI, SEA and SWAP for 72 h, followed by intracellular cytokine staining. After exclusion of doublets and dead cells identified with the live/dead marker, the CD3^+^ population (total T cells) were further classified into CD4^+^ and CD8^+^ subsets. Total splenocytes, CD4^+^ T cells and CD8^+^ T cells isolated from each spleen were determined (**a**) and the proportion of IFNγ and IL-4 producing splenic CD4^+^ or CD8^+^ T cells were determined after stimulation with (**b**) rSjLD1; (**c**) rSjTPI; (**d**) SWAP; and (**e**) SEA (* *p* value ≤ 0.05; ** *p* value ≤ 0.001; *** *p* value ≤ 0.0001).

The numbers of splenocytes and the numbers of CD4^+^ and CD8^+^ T cells isolated from spleens in all groups were determined and are shown in Figure 2a. When stimulated with rSjLD1, production of IFNγ in splenic CD4^+^ T cells isolated from rSjLD1-ISA vaccinated mice increased (*p* = 0.01) compared with control mice (Figure 2b). There was also increased production of IFNγ (*p* = 0.006) and IL-4 (*p* = 0.02) in splenic CD4^+^ T cells and IL-4 in CD8^+^ T cells (*p* = 0.03) isolated from mice immunized with rSjTPI + rSjLD1-ISA, compared with controls. After stimulation with rSjTPI (Figure 2c), significantly enhanced production of IFNγ and IL-4 in CD4^+^ T cells and IFNγ in CD8^+^ T cells was observed in splenocytes isolated from mice immunized with rSjTPI-ISA (*p* = 0.009; *p* = 0.002; *p* = 0.006) and rSjLD1 + rSjTPI-ISA (*p* = 0.0001; *p* = 0.0003; *p* < 0.0001) compared with those from control mice. In addition, stimulation with rSjTPI also induced IL-4 production in splenic CD8^+^ T cells (*p* = 0.0016) isolated from mice immunized with rSjTPI + rSjLD1-ISA, compared with cells from control mice. When stimulated with native SWAP (Figure 2d), IFNγ production in splenic CD4^+^ and CD8^+^ T cells was increased in mice vaccinated with rSjLD1-ISA (*p* = 0.01; *p* = 0.007) and rSjLD1 + rTPI-ISA (*p* = 0.02; *p* = 0.042) compared with those in the control group. No significant change in IL-4 expression in splenic CD4^+^ and CD8^+^ T cell was evident from all other groups. When stimulated with SEA (Figure 2e), only IFNγ production in splenic CD4^+^ T cells was increased (*p* = 0.03) in mice vaccinated with rSjLD1-ISA compared with the control group. The flow cytometric quantification of IFNγ- and IL-4-producing splenic CD3^+^CD4^+^ and CD3^+^CD8^+^ T cells recovered from vaccinated and control mice 6 weeks after challenge is shown in Figure S3.

##### Cytokine Responses in Mice Vaccinated with rSjLD1, rSjTPI and rSjLD1 + rSjTPI Adjuvanted with QuilA

**Figure 3:**
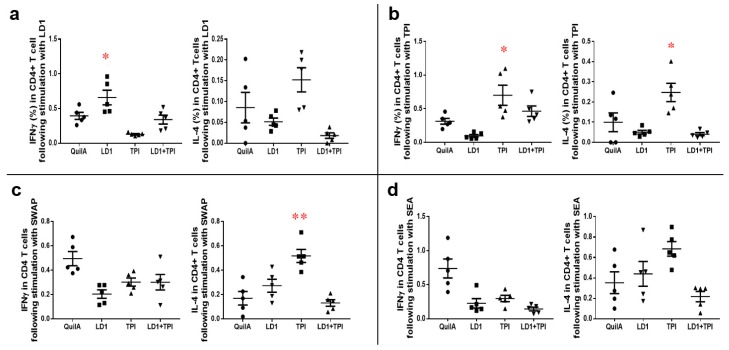
Cytokine profiles of splenic CD4^+^ T cells recovered from mice vaccinated with SjLD1, SjTPI and SjLD1 + SjTPI adjuvanted with QuilA at 6 weeks post-challenge. Splenocytes isolated from vaccinated and adjuvant control mice were stimulated with media, rSjLD1, rSjTPI, SEA and SWAP for 72 h, followed by intracellular cytokine staining. The proportion of IFNγ- and IL-4-producing splenic CD4^+^ T cells were determined after stimulation with (**a**) rSjLD1; (**b**) rSjTPI; (**c**) SWAP and (**d**) SEA (* *p* value ≤ 0.05; ** *p* value ≤ 0.001).

Following stimulation with rSjLD1 ex vivo, splenic CD4^+^ T cells isolated from mice immunized with rSjLD1 generated a significantly increased level of IFNγ (*p* = 0.047) compared with QuilA adjuvant control mice (Figure 3a). When stimulated with rSjTPI, significantly increased production of IFNγ (*p* = 0.048) and IL-4 (*p* = 0.037) was observed in splenic CD4^+^ T cells isolated from mice immunized with this protein compared with cells from the control mice (Figure 3b). However, when stimulated with SWAP, increased IL-4 production (*p* = 0.006) only occurred in splenic CD4^+^ T cells isolated from mice immunized with rSjTPI (Figure 3c), while ex vivo incubation with SEA failed to induce a significant increase in IFNγ or IL-4 production in splenic CD4^+^ T cells from vaccinated or control mice (Figure 3d). No significant production of IFNγ or IL-4 was detectable from splenic CD8^+^ T cells isolated from vaccinated or control mice (data not shown). The representative flow cytometric plots of IFNγ and IL-4-producing CD3^+^CD4^+^ splenic T cells recovered from vaccinated and control mice at 6 weeks post-challenge are shown in Figure S4.

##### Ratio of Th1/Th2 in Splenic CD4+ T Cells Harvested from Mice Vaccinated with rSjLD1, rSjTPI and rSjLD1 + rSjTPI, when Stimulated with rSjLD1, rSjTPI, SWAP or SEA

**Figure 4:**
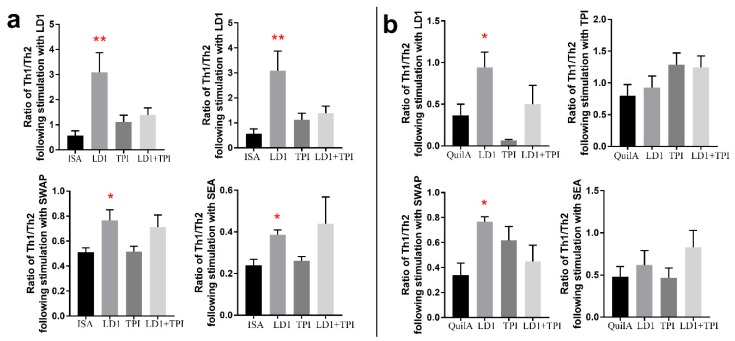
Ratio of Th1/Th2 in splenic CD4^+^ T cells obtained from mice vaccinated with SjLD1, SjTPI and SjLD1 + SjTPI adjuvanted with ISA or QuilA at 6 weeks post-challenge. The ratio of Th1/Th2 was determined by the proportion of IFNγ- and IL-4-producing splenic CD4^+^ T cells isolated from mice vaccinated with SjLD1, SjTPI and SjLD1 + SjTPI adjuvanted with (**a**) ISA and (**b**) QuilA, respectively, after stimulation with rSjLD1, rSjTPI, SWAP and SEA (* *p* value ≤ 0.05; ** *p* value ≤ 0.001).

To determine whether a Th1 or Th2 response was predominant in vaccinated mice at 6 weeks post-challenge, the ratio of Th1/Th2 was calculated using the number of IFNγ-producing splenic CD4^+^ T cells divided by the number of IL-4-producing splenic CD4^+^ T cells in individual mice in each group. We found the ratio of Th1/Th2 in splenic CD4^+^ T cells from mice vaccinated with either rSjLD1-ISA or rSjLD1-QuilA was significantly higher than that in adjuvant control mice (Figure 4) when stimulated with rSjLD1 or SWAP. An increased ratio of Th1/Th2 in splenic CD4^+^ T cells isolated from mice vaccinated with rSjLD1-ISA was also observed when incubated with SEA (Figure 4a). Those results indicate that immunisation mice with rSjLD1 conjugated with either ISA or QuilA both can elicit a predominantly Th1 response against schistosome infection.

### 2.2. The rSjLD1 Vaccine Had No Effect on Host Glucose Metabolism

**Figure 5:**
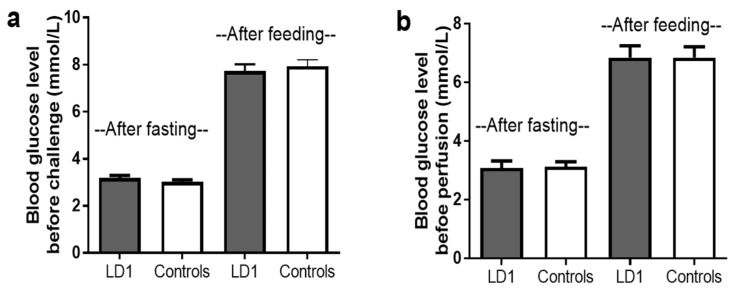
Blood glucose levels of mice were monitored while fasting (no food given overnight) and 2 h after given a meal (**a**) following the 3rd vaccination with rSjLD1 or adjuvant controls and (**b**) at 6 weeks post-challenge (before worm perfusion).

To determine the safety of the rSjLD1 vaccine and to ensure the protein had no effect on host endocrine function in vivo, blood glucose levels of mice vaccinated with rSjLD1 (just prior to cercarial challenge) during the vaccine trial, were measured for signs of diabetes when mice were subjected to overnight fasting and 2 h after feeding (Figure 5). No significant difference in blood glucose levels occurred either after fasting or after feeding between the rSjLD1-vaccinated mice and adjuvant controls indicating that the immune response elicited by the rSjLD1 vaccine did not interfere with host insulin receptor function.

### 2.3. No Immunological Cross Reactivity Occurs between Human TPI and SjTPI on the Surface of Human Cells

**Figure 6:**
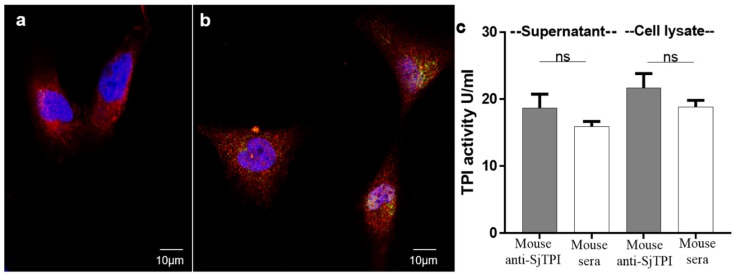
Immunofluorescence staining of SjTPI in hepatic stellate cells (HSC). Permeabilized HSC, after blocking with DAKO, were incubated with rabbit anti-actin antibody mixed with (**a**) naïve mouse serum or (**b**) mouse anti-rSjTPI serum. HSC from A and B were then incubated with a mixture of goat anti-mouse Alexa-488 (labeled with green fluorescence dye) and goat anti-rabbit Alexa-647 (labeled with red fluorescence dye). HSC nuclei were stained blue with DAPI. (**c**) TPI activity assays of supernatants and HSC lysates after culture in DMEM containing 5% (*v/v*) mouse anti-SjTPI serum or naïve mouse serum.

To determine if immunological cross reactivity occurs between human TPI and SjTPI on the surface of human cells, immunofluorescence staining of SjTPI in hepatic stellate cells (HSC) was performed using an anti-rSjTPI antibody labelled with green dye (Alexa-488) and an anti-actin antibody labeled with red dye (Alexa-647). The labeled anti-rSjTPI antibody did not bind to the surface of HSC, with minimal staining inside the HSC (Figure 6b), compared with actin staining which was distributed throughout the cells (Figure 6a). To determine whether the anti-SjTPI serum modified the TPI activity of host cells, HSC were cultured overnight in DMEM medium containing either 5% (*v/v*) mouse anti-SjTPI serum or 5% (*v/v*) normal mouse serum. The supernatants were collected and HSC cell lysates were prepared to measure TPI activity using a Colorimetric Assay Kit (BioVision, Milpitas, CA, USA). There was no significant difference in TPI activity between HSC cultured with anti-SjTPI or normal mouse serum, either in supernatants or cell lysates (Figure 6c). These data support a previous study showing that SjTPI does not trigger an auto-immune response against host TPI [15].

## 3. Discussion

Given praziquantel is the only drug available currently for the treatment of schistosomiasis, there is a pressing need to develop anti-schistosome vaccines. Unlike the African schistosomes, the zoonotic transmission of schistosomiasis japonica complicates control but it offers a unique approach for preventing human infection through the development of a transmission-blocking veterinary product. Our previous studies have demonstrated that vaccination of mice with the insulin binding domains of the SjIR1 and 2 (SjLD1 and 2 with high cross immunological reactivity) fusion proteins conferred up to 67% reduction in faecal eggs and stunting of adult worms in mice [9,16]. However, selection of a suitable adjuvant to stimulate a relevant immune response and to increase protective efficacy of candidate vaccine antigens is important in the development and employment of a successful anti-schistosome vaccine. In this study, we compared two adjuvants, ISA720 and QuilA, conjugated with rSjLD1 and showed the level of faecal egg reduction (68%) induced by vaccination of mice with rSjLD1, formulated with ISA720, and adult worm reduction (44%) induced by vaccinating mice with rSjLD1-QuilA, exceeded the 40% protective efficacy threshold established by the WHO Special Programme for Research and Training in Tropical Diseases (TDR/WHO) [3], and is comparable to the protection generated by RA (radiation attenuated) vaccines [17]. Furthermore, a significant reduction in intestinal egg burden (46–48%) and a decrease in the number of mature intestinal eggs (58–63%) observed in mice vaccinated with rSjLD1 conjugated with QuilA or ISA720 would result in consequent decreased transmission by reducing the number of eggs passing from the mesenteric veins to the intestinal lumen and thence to the external environment. By comparing two different adjuvants conjugated with SjLD1, our study further reinforced our previous findings that SjLD1 is an encouraging veterinary transmission blocking vaccine candidate [16]; however, there was no enhanced protection induced by a combination of SjLD1 and SjTPI compared with that achieved by using the SjLD1 protein alone. Importantly, we found no significant difference in blood glucose levels either after fasting or after feeding between rSjLD1-vaccinated and unvaccinated, control mice, indicating rSjLD1 is safe and has no effect on host insulin receptor function. This observation supports our previous immunoblotting analysis which revealed that there was no immunological cross—reactivity between the human insulin receptor and the SjLDs [16]. Given the fact that the mouse insulin receptor (IR) has high amino acid sequence identity with water buffalo (92%) and human (94%) IRs, we conclude that the rSjLD1 vaccine would be safe for immunising bovines and humans. 

Previous studies have demonstrated a key role for IgG antibodies in protection against schistosomiasis [18] and showed that high IgG titers before cercarial challenge correlated with low schistosome worm burdens in the olive baboon and rhesus macaque models [19]. Vaccination of mice with rSjLD1-QuilA resulted in higher levels of anti-rSjLD1 specific IgG antibodies (1:1,638,400 before challenge and 1:409,600 6-week post challenge) and increased worm burden reduction (44%) against a challenge infection than that (30%) observed in mice vaccinated with rSjLD1-ISA. It is noteworthy that the anti-rSjLD1 IgG1 and IgG2a antibodies in mice vaccinated with rSjLD1-QuilA were maintained at similar levels prior to and following cercarial challenge in the 6–12 weeks post-primary vaccination (Figure 1). In contrast, both the IgG1 and IgG2a responses elicited in mice vaccinated with rSjLD1-ISA declined post-cercarial challenge, reaching the lowest titre just prior to perfusion 6 weeks post-challenge. This suggests that the QuilA adjuvant can help generate high levels of IgG1 and IgG2a in mice which may play a key role in reducing worm survival and worm development during the early stages of infection. It is important to stress that, in this study, we measured IFNγ and IL-4 levels in splenic CD4^+^ T cells isolated from vaccinated mice at 6 weeks post-challenge when *S. japonicum* worms are fully mature and have already laid considerable numbers of eggs. In many previous studies, analysis of the immune response was performed immediately after the completion of the vaccination regimen and prior to cercarial challenge, and the results obtained indicated the protective immune response induced by vaccination was associated with a predominant Th1 response [20,21]. At 6-week post-challenge, we found significantly enhanced IFNγ levels with increased ratio of Th1/Th2 (Figure 4) were produced in splenic CD4^+^ T cells of both rSjLD1-ISA (Figure 2b) and rSjLD1-QuilA (Figure 3a) vaccinated mice when stimulated with rSjLD1, reflecting the increased Th1 response after being inhibited by a strong Th2 response elicited by newly-laid eggs. These data add further support to a previous study showing that a high specific IFN-γ (Th1) response was associated with a high level of protection against schistosome infection in mice immunised with the RA vaccine [22]. However, the production of IFNγ in splenic CD4^+^ T cells isolated from rSjLD1-QuilA-vaccinated mice, following SWAP and SEA stimulation, was decreased which is the result of an increased Th2 response elicited by newly-laid eggs. In contrast, immunisation of mice with rSjLD1-ISA induced higher reductions in liver eggs (56%), intestinal eggs (48%) and fecal egg (68%) compared with mice vaccinated with rSjLD1-QuilA (Table 1). This was also reflected by the consistently increased levels of IFNγ in splenic CD4^+^ T cell isolated from rSjLD1-ISA-vaccinated mice at 6 weeks post-challenge, when stimulated with rSjLD1, SWAP or SEA (Figure 2). 

Immunisation of mice with rSjTPI-ISA rSjTPI-QuilA resulted in strong anti-rSjTPI-specific IgG production (1:204,800–1:819,200) and contributed to enhanced production of IFNγ and IL-4 in splenic CD4^+^ T cells (i.e., a mixed Th1/Th2 response) when stimulated with rSjTPI. However, the immunological response generated did not achieve a significant level of protection against *S. japonicum* infection, an outcome similar to that reported by another recent study in mice [23]. In contrast, a SjTPI DNA priming-protein boosting vaccine strategy was shown to generate a high level of protective efficacy in BALB/c mice [24,25]. Following stimulation with SWAP and SEA, decreased production of IFN-γ and increased IL-4 in splenic CD4^+^ T cells isolated from mice vaccinated with rSjTPI-QuilA was observed compared with adjuvant controls, indicating the induction by adult worms and new-laid eggs at 6 weeks post-cercarial challenge of an increased Th2 response or the switch to a Th2 response, resulting in a lack of protective efficacy following immunisation with rSjTPI.

Much of our current knowledge of schistosome immunology and the mechanism of pathogenesis in schistosomiasis has been established through studies conducted in mice [26,27,28]. The murine model of schistosomiasis has also been used extensively in vaccine development through the testing of vaccine candidates for protective efficacy [29,30,31]. The advantages of using mice to pre-screen vaccine candidates include: (1) substantially lower cost (e.g., the cost/animal in using non-human primates or, bovines, in the case of *S. japonicum*, are considerably higher); (2) the ability to test multiple vaccine formulations on statistically robust numbers of animals; (3) better controlled laboratory conditions; and (4) the availability of large panels of reagents to measure and characterise protective immune responses. To date, the majority of schistosomiasis vaccine data have been obtained with mice. However, the use of mice to test schistosome vaccine candidates has come under recent scrutiny and it has been suggested that the murine model may be inherently flawed resulting in inaccurate protective efficacy data [32]. In the case of schistosomiasis japonica it is thus critical to translate data obtained in the laboratory mouse model to relevant, natural *S. japonicum* hosts such as water buffaloes [33] which are major reservoirs, critically involved in disease transmission to humans, in a controlled setting. In addition, the immunological responses of bovines tend to be more comparable to human responses than those of mice in the differentiation of Th1/Th2 responses to schistosome antigens [33,34]. These and other aspects of bovine immunology, as they relate to the responses to *S. japonicum* vaccine antigens, need to be further investigated [35]. 

## 4. Materials and Methods

### 4.1. Ethics Statement

The conduct and procedures involving animal experimentation were approved by the Animal Ethics Committee of QIMR Berghofer Medical Research Institute (project number 288 and ethics ID A0108-054, approval date 31 October 2017). This study was performed in accordance with the recommendations in the Guide for the Care and Use of Laboratory Animals of the USA National Institutes of Health.

### 4.2. Parasites

*Oncomelania hupensis hupensis* snails (Anhui Province isolate, China) infected naturally with *S. japonicum*, were transported to QIMR Berghofer, Australia. Cercariae, freshly shed from infected snails, were collected as reported [36].

### 4.3. Protective Efficacy of the Recombinant SjLD1 and SjTPI Vaccine

#### 4.3.1. *Escherichia coli* Protein Expression 

A 1065 bp fragment of cDNA encoding SjLD1 [16] and a 714 bp fragment of cDNA encoding SjTPI [37], were amplified by PCR and inserted into the pET28b vector (Invitrogen, Carlsbad, CA, USA), respectively. *E. coli* BL21 (DE3) cells (Invitrogen) were transformed with the reconstructed plasmids for protein expression as described [9]. The expressed SjTPI and SjLD1 proteins were purified from *E. coli* lysates using Ni-NTA affinity chromatography (GE Health Life Science, Pittsburgh, PA, USA). Recombinant SjLD1 (rSjLD1) protein was further purified (denaturing conditions; 6 M guanidine-HCl) and refolded in buffer (300 mM NaCl, 50 mM NaH_2_PO_4_, 8% *w*/*v* sucrose, PH 4.5). Purified proteins had residual endotoxin removed [9] and Endotoxin (*E. coli*) Standards kits (Lonza, Basel, Switzerland) were used subsequently to assess any contamination. The purified rSjLD1 and rSjTPI proteins were used in animal vaccine/challenge experiments and for immunological analysis.

#### 4.3.2. Animal Immunization and Challenge Experiments

Two independent vaccination-challenge trials (trials 1, 2) with purified rSjLD1 and rSjTPI, involving four groups of female CBA mice (6–8 weeks old, 10 mice/group) per trial, were undertaken. Trial 1: Adjuvant control mice received subcutaneously (s.c.) 100 μL of PBS homogenised with 15 µg of Quil A adjuvant (InvivoGen, San Diego, CA, USA). Mice in the three vaccinated groups were immunised s.c. with 25 µg of rSjLD1, 25 µg rSjTPI or 25 µg rSjLD1 + rSjTPI proteins, respectively, in 100 µL of PBS homogenised with 20 µg of Quil A adjuvant. Trial 2: Adjuvant control mice received s.c. 50 μL of a 70% volume formulation of Montanide™ ISA 720 VG (SEPPIC, Paris, France) [31]. Mice in the three vaccinated groups were immunised s.c. with 25 µg of rSjLD1, 25 µg rSjTPI or 25 µg rSjLD1 + rSjTPI (with equal amounts of rSjLD1 and SjTPI) proteins, respectively, in 50 μL of a 70% volume formulation of ISA 720 VG. In both trials the mice were boosted twice at 2 weeks intervals using the same vaccine regimen. All mice were challenged with 34 ± 1 *S. japonicum* cercariae percutaneously via the abdomen 2 weeks after the third injection.

#### 4.3.3. Worm and Egg Counting and Pathology 

The numbers of *S. japonicum* worms and eggs in faeces, intestines and livers were determined [9] in the vaccinated and control mice at 6 weeks post-cercarial challenge to determine the vaccine efficacy of rSjTPI, rSjLD1, and rSjLD1 + rSjTPI. Adult worms were counted and all parasites per mouse were fixed and their lengths determined [9]. Faecal samples were collected from each mouse individually in all the groups 2 days prior to perfusion to assess the faecal egg output [9]. The left anatomical lobes of the livers from each of the mice were fixed in 4% (*v/v*) formalin, paraffin-embedded sections were made and stained with H&E (haematoxylin and eosin). All slides were digitised using an Aperio Slide Scanner (Leica Biosystems Inc, Buffalo Grove, IL, USA) and liver pathology was assessedby measuring the volume density of granulomatous lesions using Aperio ImageScope v11.1.2.760 software (Leica Biosystems Inc, Buffalo Grove, IL, USA). 

#### 4.3.4. Specific Antibody Responses

Sera were collected at 0, 2, 4, 6, 8, 10, 12 and 14 weeks post-first immunisation to determine by ELISA IgG, IgG1, IgG2a, IgG2b, IgG2c and IgG3 antibody responses in the rSjLD1/rSjTPI vaccinated mice [9]. In brief, rSjLD1 or rSjTPI fusion proteins (10 μg/mL, 100 μL/well) were used to coat immunoplates (Nalge Nune International, New York, NY, USA) at 4 °C overnight and then each well was blocked with blocking buffer (1% BSA in PBST) at 37 °C for 1 h. Serum samples were diluted serially with blocking buffer (100 μL/well), added to the wells and the plates incubated for 1 h at 37 °C HRP-conjugated sheep anti-mouse IgG, IgG1, IgG2a, IgG2b, IgG2c and IgG3 antibodies (Invitrogen) were added (1:2000, 100 μL/well) and the plates incubated at 37 °C for 1 h. Streptavidin-HRP (BD Pharmingen, San Jose, CA, USA) (1:10,000) was added (100 μL/well) to each well. Each plate was subjected to 5XPBST washes after each step, with 2 min between steps. Reactions were developed with TMB substrate (100 μL/well) for 5 min and stopped with 2 M sodium hydroxide (50 μL/well) [38]. Optical density (OD) values were read by microplate reader at 450 nm, and all tests were undertaken in duplicate on each test plate. Positive antibody responses were defined as OD values higher than 2.1 times the OD mean values of sera of mouse controls. 

#### 4.3.5. Blood Glucose Levels of SjLD1-Vaccinated Mice and Controls

It is now recognized that SjLD1 is important in activating the insulin pathway in schistosomes and may help to regulate glucose uptake by these parasites from host blood. To test whether vaccination with rSjLD1 had any effect on host blood glucose levels in vivo, blood glucose concentrations were measured in rSjLD1-vaccinated mice and QuilA and ISA adjuvant controls using a Roche Accu-Chek Performa blood glucose meter and test Strips (Roche, North Ryde, NSW, Australia) two weeks after the 3rd vaccination, when mice were fasting, and then 2 h after being fed for signs of diabetes.

#### 4.3.6. Flow Cytometry Analysis 

All mice were sacrificed after 6 weeks post-cercarial challenge. The spleens of each animal (*n* = 5/group) were obtained and splenocytes isolated [39]. In brief, spleens were pressed through 70 µm cell strainers and red blood cells lysed with RBC lysis buffer (Sigma-Aldrich, St. Louis, MO, USA). Following repeated steps of washing and centrifugation, the cells were re-suspended in IMDM (Iscoves Modified Dulbecco’s Medium) containing 10% (*v/v*) fetal bovine serum, 100 Units/mL Penicillin, 100 μg/mL Streptomycin and 2-Mercaptoethanol (0.05 mM). Cell were seeded into 96-well plates (5 × 10^5^ cells/well). The cells were incubated in the presence (each, 2 μg/well) of rSjTPI or rSjLD1, SEA (soluble egg antigen) or SWAP (soluble worm antigen preparation) for 72 h at 37 °C. ConA (0.1 μg/well) stimulation was performed as positive control and non-stimulated splenocytes were used as negative control. Brefeldin A (BFA) was added (2 µg/well) to cell cultures 6 h prior to harvesting.

The collected cells were blocked with anti-FcR (2.4G2), stained with BD Horizon™ fixable viability stain 780 (BD Biosciences, North Ryde, NSW, Australia) and labeled with anti-mouse CD8 BV650, CD4-Alexa Fluor700 and CD3-BV421 antibodies. Cells were fixed and permeabilised with BD cytofix/cytoperm buffer as per the manufacturer’s protocol and stained for intracellular cytokine expression using anti mouse IFNγ-PerCp Cy5.5, IL-4 PE CF594 (BD Biosciences); the corresponding fluorescence minus one controls were also prepared. Samples were then collected on a BD Fortessa 4 Laser flow cytometer using FACSDiva™ (BD Biosciences) and the results analyzed by Flowjo 9 software (FlowIo, LLC, Ashland, OR, USA). All statistical analysis used GraphPad Prism version 6 (GraphPad Software, La Jolla, CA, USA).

### 4.4. Immunofluorescence-Staining of Mouse Anti-SjTPI Antibody in Hepatic Stellate Cells and TPI Activity Assays

#### 4.4.1. Immunofluorescence-Staining

Hepatic stellate cells (HSC), a major cell type involved in liver fibrosis [40], were available within the department and were authenticated by Short tandem repeat (STR) profiling. HSC were cultured in DMEM (Dulbecco’s Modified Eagle Medium) (Invitrogen) plus 10% foetal calf serum (FCS), and then incubated in serum-free medium for 10 min at room temperature (RT). Cells were then fixed by 2% (*v/v*) paraformaldehyde in PBS (pH 7.3) for 5 min at RT. The fixed cells were washed with PBS and then permeabilized by 0.2% (*v/v*) Triton X-100 in PBS for 15 min [41] followed by washing with PBS and then permeabilized by 0.2% Triton X-100 in PBS for 15 min [41]. Cells were blocked with Protein Blocking Reagent (DAKO) for 20 min and then incubated with a mixture of two primary antibodies containing equal amounts (100 μL) of mouse anti-SjTPI serum diluted 1:50 in 2% BSA-PBS (pH 7.3) and 100 μL of rabbit anti-Actin antibody (1:75 diluted in 2% BSA-PBS) (Invitrogen) for 2 h at RT. Naïve mouse serum (1:50 diluted in 2% BSA-PBS) mixed with rabbit anti-actin antibody was used as negative control. Following a wash with PBS, cells were incubated in a mixture of equal amounts of goat anti-mouse Alexa-488 (Invitrogen) and goat anti-rabbit Alexa-647 (Invitrogen) (1:150 diluted in 2% BSA-PBS) for 30 min in the dark at RT. Cells were then stained with DAPI and ProLong Diamond Antifade Reagent (Invitrogen) was used to mount cover slips. Images were captured using a DeltaVision Deconvolution Microscope (Applied Precision, Washington, DC, USA).

#### 4.4.2. TPI Activity Assays 

HSC were cultured overnight in flasks in media containing 5% (*v/v*) naïve mouse serum, mouse anti-SjTPI antibody or FCS to determine any effect of the anti-SjTPI antibody on the host cells. Cell culture supernatants were then collected and supernatants of cell lysates prepared and used to measure TPI activity using TPI Activity Colorimetric Assay Kits (BioVision). Cell lysates were prepared by washing HSC gently with cold PBS, followed by the addition of cold RIPA lysis buffer (50 mM NaCl, 5 mM EDTA (PH 8.0), 50 mM Tris∙Cl (PH 8.0), 1% (*v/v*) NP-40, 0.5% sodium deoxycholate, 0.1% SDS), and then cells were scraped from the flask surface using a cell scraper. The HSC were incubated on ice for 15 min, then sonicated on ice, followed by an additional incubation on ice for 15 min. The cell lysate was then centrifuged at 13,000× *g* for 5 min at 4 °C and the supernatant was used in TPI activity assays.

### 4.5. Statistical Analysis

Data are presented as the mean ± SE. Group comparisons were assessed by one-way ANOVA for statistically significant differences, defined as a *p* value ≤ 0.05 using GraphPad Prism software (Version 7.02) (* *p* value ≤ 0.05; ** *p* value ≤ 0.001; *** *p* value ≤ 0.0001).

## 5. Conclusions

In summary, we have shown that vaccination of mice with rSjLD1 induced a significant reduction in the number of *S. japonicum* worms and tissue eggs, likely depressed the maturity of eggs, and a substantial decrease in faecal eggs This suggests that development of a rSjLD1-based vaccine to prevent the transmission of zoonotic schistosomiasis is achievable but that now it needs to be translated to the natural water buffalo host so as to determine whether its field application can help reduce human schistosomiasis transmission. This would have clear public health benefits with the potential for future wide-scale application in schistosomiasis-endemic areas of China and the Philippines.

## Figures and Tables

**Table 1:** Parasitologic data for vaccinated and control mice challenged with 34 ± 1 *Schistosoma japonicum* cercariae.

Adjuvant	Group	Number Adult Worms Mean ± SE	Mean Length of Adult Worms (mm) Mean ± SE %Reduction (*p* Value)	Liver Eggs/g Mean ± SE %Reduction (*p *Value)	Intestinal Eggs/g Mean ± SE %Reduction (*p* Value)	Maturity of Intestinal Eggs in Stage V (%) Mean ± SE %Reduction (*p* Value)	Faecal Eggs/g/f Mean ± SE %Reduction (*p* Value)
QuilA.	Control *n* = 10	(F) 5.9 ± 0.7 (M) 8.5 ± 0.8	(F) 10.4 ± 0.2 (M) 6.6 ± 0.2	41886 ± 6001	64483 ± 9998	21.6 ± 2	1398 ± 474
	SjLD1 *n* = 10	(F) 3.3 ± 0.6 44% ** (*p *= 0.008) (M) 4.8 ± 0.8 43.5% * (*p *= 0.03)	(F) 10.8 ± 0.3 (M) 7.7 ± 0.3	23285 ± 4834 44% * (*p *= 0.03)	34533 ± 8038 46% * (*p *= 0.04)	8.9 ± 1.5 58% * (*p *= 0.04)	546 ± 276 61% * (*p *= 0.02)
	SjTPI *n* = 10	(F) 5.7 ± 0.6 (M) 7.4 ± 1.0	(F) 10.7 ± 0.3 (M) 8.3 ± 0.3	42894 ± 4548	55473 ± 7574 14% ns (*p *= 0.4)	4.4 ± 0.3 79% *** (*p *= 0.0002)	776 ± 354 44% ns (*p *= 0.36)
	SjLD1 + TPI *n* = 10	(F) 5.7 ± 0.9 (M) 8.2 ± 1.1	(F) 10.6 ± 0.2 (M) 7.1 ± 0.3	41649 ± 4391	61291 ± 12505	8.4 ± 2.3 67% * (*p *= 0.03)	885 ± 320 36% ns (*p *= 0.32)
ISA-720	Control *n *= 10	(F) 5.3 ± 0.9 (M) 11.1 ± 1.6	(F) 11.1 ± 0.4 (M) 6.9 ± 0.1	36625 ± 2271	78969 ± 9486	26.6 ± 1.8	4278 ± 1019
	SjLD1 *n* = 10	(F) 3.7 ± 0.7 30% * (*p *= 0.05) (M) 12.2 ± 1.1	(F) 9.5 ± 0.2 13% ** (*p *= 0.003) (M) 5.8 ± 0.1 16% *** (*p *< 0.0001)	16233 ± 3276 56% *** (*p *= 0.0003)	41331 ± 10912 48% * (*p *= 0.013)	9.9 ± 2.0 63% * (*p *= 0.01)	1349 ± 459 68% * (*p *= 0.05)
	SjTPI *n* = 10	(F) 5.8 ± 0.7 (M) 12.7 ± 1.7	(F) 9.8 ± 0.2 10% ** (*p *= 0.009) (M) 5.7 ± 0.1 18% *** (*p *< 0.0001)	28785 ± 3306 21% ns (*p *= 0.08)	74104 ± 10278	8.6 ± 1.1 68% *** (*p *= 0.0007)	2089 ± 410 51% ns (*p *= 0.06)
	SjLD1 + TPI *n* = 10	(F) 5.8 ± 1.1 (M) 12.8 ± 1.4	(F) 8.7 ± 0.2 20% *** (*p *< 0.0001) (M) 5.4 ± 0.1 22% *** (*p *< 0.0001)	24735 ± 3558 33% * (*p *= 0.03)	53788 ± 10381 32% * (*p *= 0.05)	11.8 ± 0.9 56% * (*p *= 0.06)	2158 ± 399 50% ns (*p *= 0.07)

F, female worm; M, male worm; *n*, the number of mice per group that survived the trial and were necropsied; ns, not significant; SE, standard error of the mean; * *p* value ≤ 0.05; ** *p* value ≤ 0.001; *** *p* value ≤ 0.0001.

## Supplementary Materials

Supplementary materials can be found at http://www.mdpi.com/1422-0067/19/10/3088/s1.
