# Supplementary material for: Protective Immune Responses Generated in a Murine Model Following Immunization with Recombinant Schistosoma japonicum Insulin Receptor
Source: Int J Mol Sci. 2018 Oct 9;19(10):3088. doi: 10.3390/ijms19103088 (PMC6213549; doi:10.3390/ijms19103088)
Supplement: Supplementary file 1 [file ijms-19-03088-s001.zip › ijms-361357-SI.pdf]

**Figure S1**. The recombinant proteins SjLD1 and SjTPI and the combination of SjLD1 and SjTPI, formulated with QuilA, induced reductions in worm burden **(a, b, c)**, liver eggs **(d)**, intestinal eggs **(e)**, maturity of intestinal eggs **(f)** and faecal eggs **(g)** in mouse vaccine/challenge trials. *P* value *=≤0.05, **=≤0.01

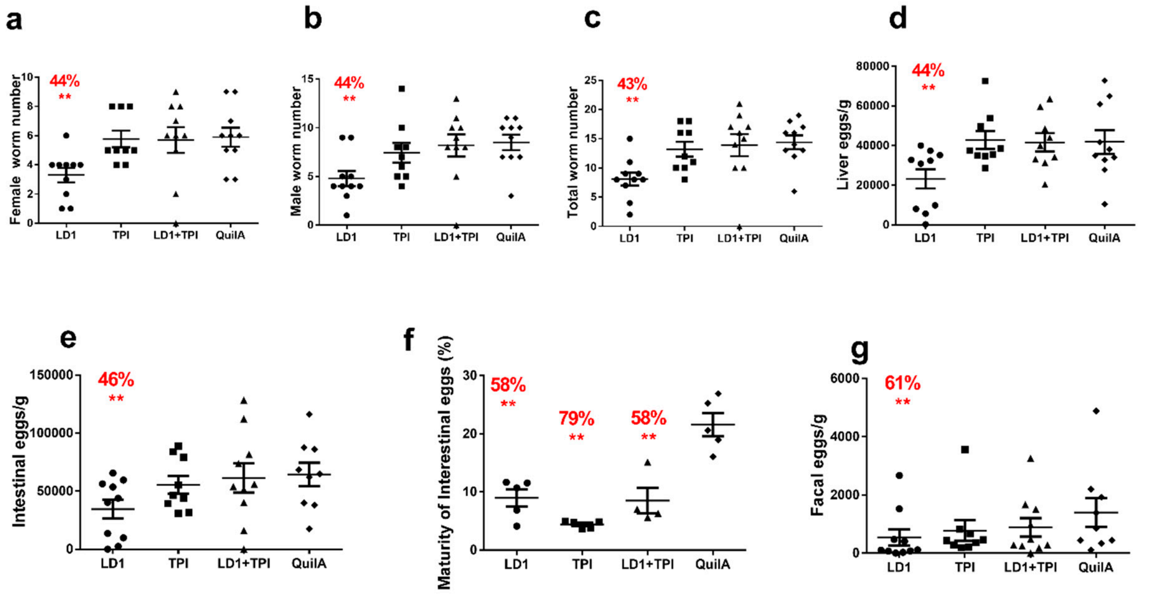

**Figure S2**. The recombinant proteins SjLD1 and SjTPI and the combination of SjLD1 and SjTPI, formulated with ISA induced reductions in worm burden **(a, b, c),** length of worms **(d, e)** and liver eggs **(f)**, intestinal eggs **(g)**, maturity of intestinal eggs **(h)** and faecal eggs **(i)** in mouse vaccine/challenge trials. *P* value *=≤0.05, **=≤0.01, ***=≤0.001

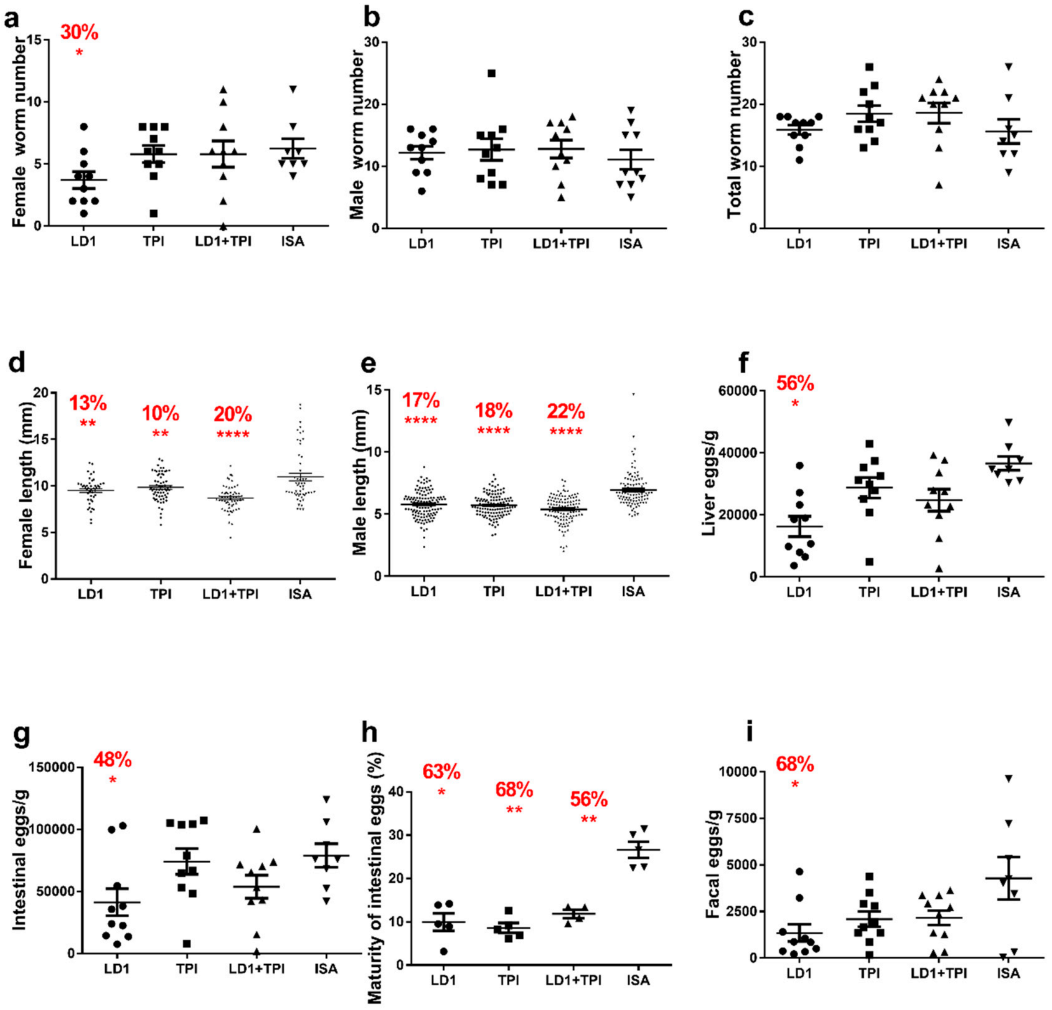

**Figure S3**. The flow cytometric quantification of IFNγ- and IL-4-producing splenic CD3+CD4+ and CD3+CD8+ T cells recovered from mice vaccinated with recombinant proteins SjLD1, SjTPI, SJLD1+SjTPI conjugated with ISA and control mice injected with ISA, 6 weeks after challenge.

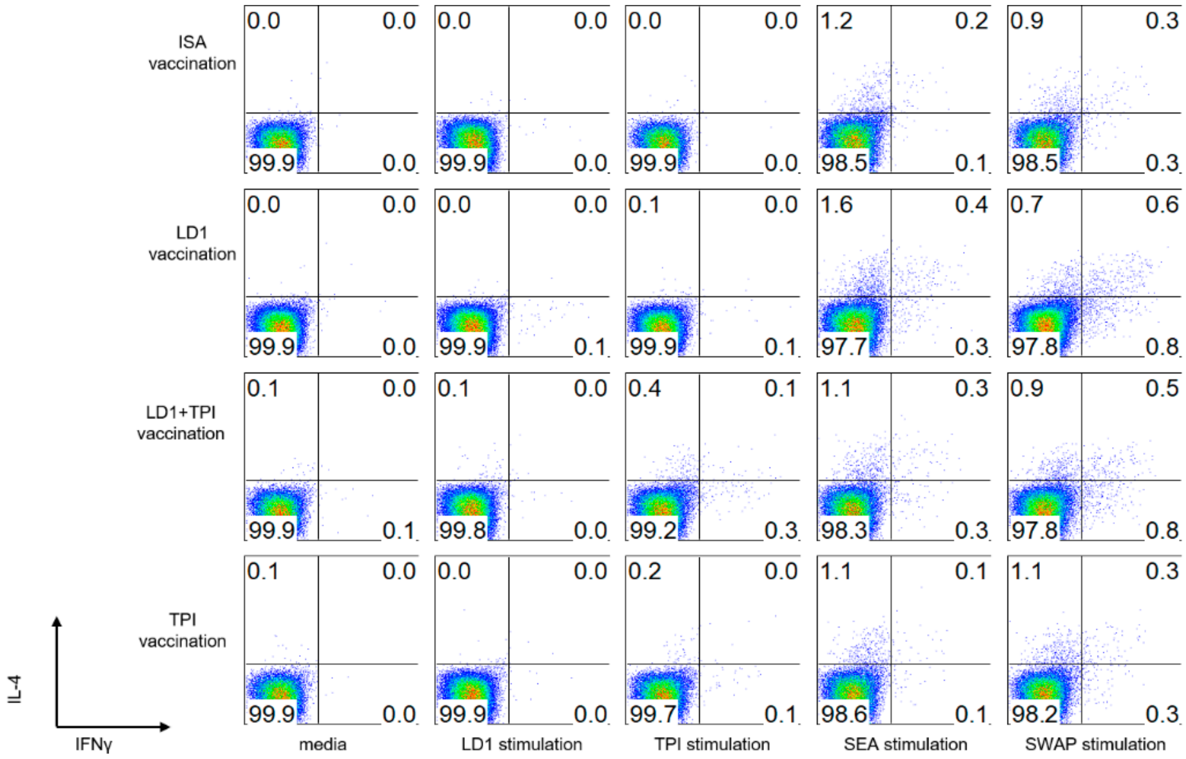

**Figure S4.** The flow cytometric quantification of IFNγ- and IL-4-producing splenic CD3$^+$CD4$^+$ and CD3$^+$CD8$^+$ T cells recovered from mice vaccinated with recombinant proteins SjLD1, SjTPI, SJLD1+SjTPI conjugated with QuilA and control mice injected with QuilA, 6 weeks after challenge.

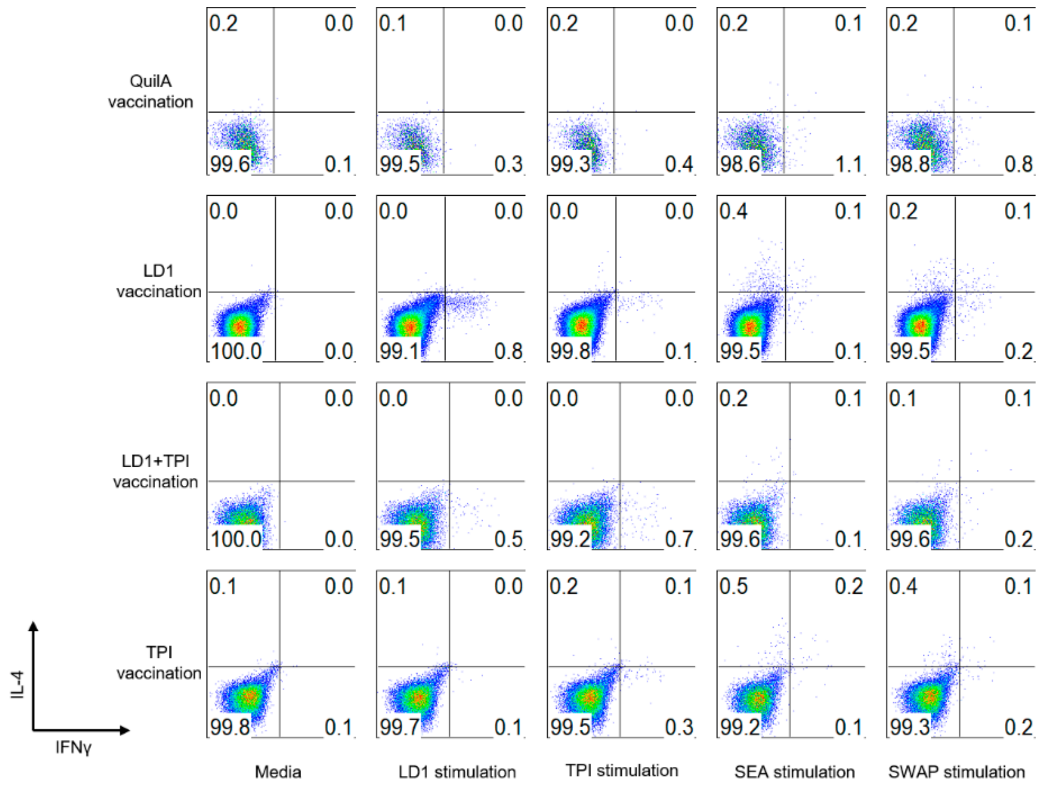

**Table S1.** Titres of IgG antibody subtypes in sera of mice vaccinated with rSjLD1, rSjTPI and rSjLD1+rSjTPI before challenge and before perfusion.

| Adjuvant | Titre of IgG | Before Challenge | | | | Before Perfusion | | | | Control group |
|---|---|---|---|---|---|---|---|---|---|---|
| | | rSjLD1 group | rSjTPI group | rSjLD1+rSjTPI group | | rSjLD1 group | rSjTPI group | rSjLD1+rSjTPI group | | |
| | | | | Against rSjLD1 | Against rSjTPI | | | Against rSjLD1 | Against rSjTPI | |
| **QuilA** | IgG | 1:1,638,400 | 1:204,800 | 1:819,200 | 1:819,200 | 1:409,600 | 1:51,200 | 1:204,800 | 1:204,800 | 1:50 |
| | IgG1 | 1:409,600 | 1:102,400 | 1:409,600 | 1:409,600 | 1:102,400 | 1:51,200 | 1:51,200 | 1:51,200 | 1:50 |
| | IgG2a | 1:819,200 | 1:204,800 | 1:409,600 | 1:409,600 | 1:102,400 | 1:51,200 | 1:51,200 | 1:102,400 | 1:50 |
| | IgG2b | 1:409,600 | 1:204,800 | 1:204,800 | 1:409,600 | 1:102,400 | 1:51,200 | 1:25,600 | 1:51,200 | 1:50 |
| | IgG2c | 1:51,200 | 1:204,800 | 1:51,200 | 1:204,800 | 1:6,400 | 1:25,600 | 1:12,800 | 1:25,600 | 1:50 |
| | IgG3 | 1:12,800 | 1:6,400 | 1:12,800 | 1:12,800 | 1:6,400 | 1:3,200 | 1:6,400 | 1:6,400 | 1:50 |
| **ISA** | IgG | 1:819,200 | 1:819,200 | 1:409,600 | 1:1,638,400 | 1:102,400 | 1:409,600 | 1:51,200 | 1:409,600 | 1:50 |
| | IgG1 | 1:204,800 | 1:819,200 | 1:204,800 | 1:819,200 | 1:25,600 | 1:204,800 | 1:51,200 | 1:204,800 | 1:50 |
| | IgG2a | 1:204,800 | 1:204,800 | 1:6,400 | 1:204,800 | 1:51,200 | 1:51,200 | 1:1,600 | 1:25,600 | 1:50 |
| | IgG2b | 1:20,4800 | 1:102,400 | 1:6,400 | 1:102,400 | 1:51,200 | 1:12,800 | 1:3,200 | 1:12,800 | 1:50 |
| | IgG2c | 1:25,600 | 1:51,200 | 1:3,200 | 1:51,200 | 1:3,200 | 1:25,600 | 1:800 | 1:25,600 | 1:50 |
| | IgG3 | 1:12,800 | 1:6,400 | 1:1,600 | 1:6,400 | 1:6,400 | 1:6,400 | 1:1,600 | 1:3,200 | 1:50 |

**Table S2.** Specific anti-SjLD1 and anti-SjTPI IgG1 and IgG2a immune profiles in mice induced by vaccinated with recombinant SjLD1 or recombinant SjTPI, at different time points after the first vaccination.

| Weeks after first vaccination | SjLD1 | | | | | | SjTPI | | | | | |
|---|---|---|---|---|---|---|---|---|---|---|---|---|
| | QuilA | | | ISA | | | QuilA | | | ISA | | |
| | IgG | IgG1 | IgG2a | IgG | IgG1 | IgG2a | IgG | IgG1 | IgG2a | IgG | IgG1 | IgG2a |
| 0 | 0.15±0.004 | 0.14±0.003 | 0.1±0.002 | 0.12±0.003 | 0.12±0.002 | 0.11±0.003 | 0.15±0.002 | 0.12±0.003 | 0.17±0.002 | 0.16±0.0005 | 0.12±0.003 | 0.145±0.003 |
| 2 | 0.42±0.05 | 0.31±0.04 | 0.2±0.05 | 0.41±0.03 | 0.15±0.03 | 0.10±0.01 | 0.36±0.02 | 0.19±0.02 | 0.24±0.006 | 0.46±0.05 | 0.35±0.018 | 0.10±0.002 |
| 4 | 1.01±0.06 | 0.52±0.01 | 0.56±0.07 | 0.85±0.01 | 0.18±0.06 | 0.27±0.01 | 1.21±0.06 | 1.16±0.01 | 1.13±0.019 | 1.40±0.03 | 0.86±0.014 | 0.29±0.022 |
| 6 | 1.81±0.06 | 0.72±0.05 | 0.68±0.06 | 1.6±0.05 | 0.55±0.28 | 0.59±0.06 | 1.52±0.06 | 1.25±0.03 | 1.17±0.05 | 1.71±0.06 | 0.91±0.0075 | 0.34±0.05 |
| 8 | 1.62±0.04 | 0.69±0.05 | 0.67±0.02 | 1.5±0.05 | 0.61±0.14 | 0.59±0.06 | 1.33±0.05 | 1.18±0.06 | 1.15±0.066 | 1.59±0.07 | 0.93±0.06 | 0.39±0.064 |
| 10 | 1.55±0.08 | 0.70±0.03 | 0.67±0.08 | 1.3±0.07 | 0.54±0.19 | 0.51±0.08 | 1.29±0.06 | 1.16±0.002 | 1.10±0.069 | 1.58±0.08 | 0.84±0.022 | 0.32±0.08 |
| 12 | 1.43±0.07 | 0.60±0.06 | 0.63±0.06 | 1.2±0.06 | 0.28±0.09 | 0.28±0.07 | 1.22±0.07 | 1.11±0.073 | 1.09±0.053 | 1.55±0.08 | 0.76±00073 | 0.29±0.093 |
